# Case report: a rare clinical presentation of a difficult diagnosis of dedifferentiated liposarcoma showing leiomyosarcoma phenotype in the ileocecal region

**DOI:** 10.3389/fonc.2024.1425809

**Published:** 2024-11-11

**Authors:** Tomonori Kawasaki, Tomoaki Tashima, Kojiro Onohara, Yasumitsu Hirano, Misuzu Yamato, Suguru Shirotake, Tomoaki Torigoe, Yasuo Yazawa, Masataka Hirasaki, Masanori Wako, Taro Fujimaki, Jiro Ichikawa

**Affiliations:** ^1^ Department of Pathology, Saitama Medical University International Medical Center, Saitama, Japan; ^2^ Department of Gastroenterology, Saitama Medical University International Medical Center, Saitama, Japan; ^3^ Department of Radiology, Interdisciplinary Graduate School of Medicine, University of Yamanashi, Yamanashi, Japan; ^4^ Department of Gastroenterological Surgery, Saitama Medical University International Medical Center, Saitama, Japan; ^5^ Department of Uro-Oncology, Saitama Medical University International Medical Center, Saitama, Japan; ^6^ Department of Orthopedic Oncology & Surgery, Saitama Medical University International Medical Center, Saitama, Japan; ^7^ Department of Clinical Cancer Genomics Saitama Medical University International Medical Center, Saitama, Japan; ^8^ Department of Orthopedic Surgery, Interdisciplinary Graduate School of Medicine, University of Yamanashi, Chuo, Japan

**Keywords:** dedifferentiated liposarcoma, imaging, immunohistochemistry, leiomyosarcoma, gastrointestinal stroma tumor

## Abstract

Dedifferentiated liposarcoma is a malignant lipomatous tumor that rarely occurs in the gastrointestinal tract, including the ileocecal region. In this case, computed tomography and magnetic resonance imaging showed no fatty mass located in the mesenteric or submucosal lesion, and positron emission tomography–computed tomography showed a high maximum standardized uptake value, collectively indicating the gastrointestinal stroma tumor and lymphoma. The pathological findings resemble leiomyosarcoma; the immunohistochemistry findings including mouse double minute 2 homolog and cyclin D-dependent kinase-4 and amplification of mouse double minute 2 homolog in fluorescence *in situ* hybridization just favored the diagnosis of dedifferentiated liposarcoma with leiomyosarcoma phenotype and not leiomyosarcoma. Recently, a new inhibitor for mouse double minute 2 homolog and cyclin D-dependent kinase-4 has been developed, and clinical trials for dedifferentiated liposarcoma are currently ongoing. This could change the treatment strategy drastically compared with other soft tissue sarcomas. Hence, a correct diagnosis of dedifferentiated liposarcoma is required.

## 1 Introduction

Dedifferentiated liposarcoma (DDLPS) generally occurs in the retroperitoneum and rarely in the gastrointestinal tract (1, 2). Approximately 10% of atypical lipomatous tumors/well-differentiated liposarcoma (ALT/WDLPS) can be dedifferentiated. ALT/WDLPS and DDLPS can reoccur; however, DDLPS has metastatic potential (3). DDLPS was suspected from imaging studies, which showed the presence of relatively high-fat components and a relatively low standardized uptake value (SUV) in positron emission tomography (PET) in the typical location. However, correctly diagnosing DDLPS by only imaging is challenging, except when using typical imaging (4, 5). DDLPS and ALT/WDLPS are characterized by high amplification of the 12q14-15 chromosomal region, containing mouse double minute 2 homolog (MDM2) and cyclin D-dependent kinase-4 (CDK4) (3). The positive finding of MDM2 and CDK4 in immunohistochemistry (IHC) and fluorescence *in situ* hybridization (FISH) contribute to the correct diagnosis of DDLPS because i) DDLPS differential diagnosis is widespread from benign to malignant tumors and ii) DDLPS rarely showed heterogeneous differentiation (3). Gastrointestinal stromal tumor (GIST) and lymphoma frequently occur from the point of occurrence in the ileocecal region. The differential diagnosis of GIST and diagnosis with pathological findings from benign and malignant soft tissue tumors, including leiomyosarcoma, are often difficult (6). Here, we report a case of DDLPS with leiomyosarcoma phenotype characterized by rare location, atypical imaging, and difficult histopathological findings.

## 2 Case report

**Figure 1:**
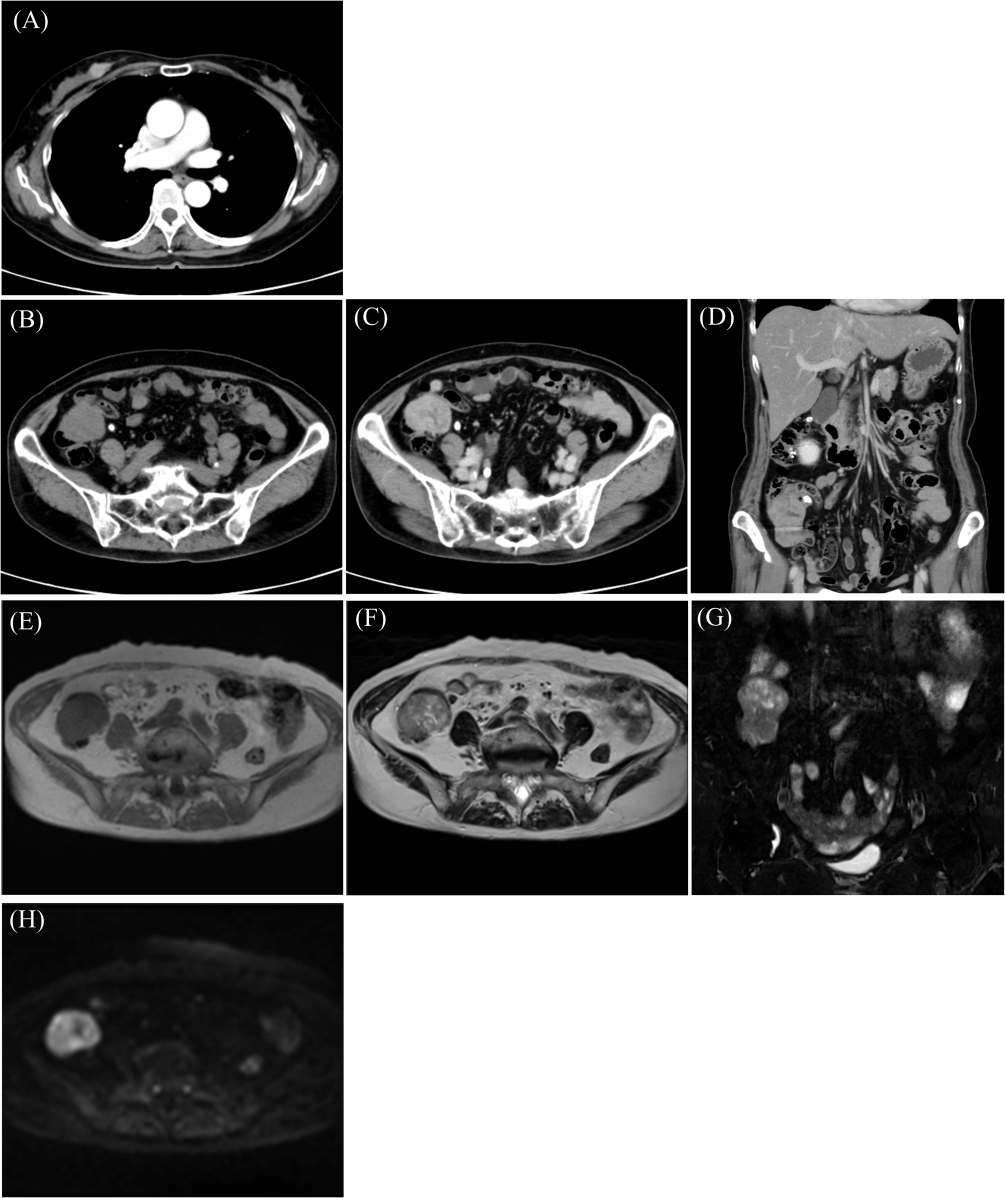
Computed tomography (CT) shows a mass with enhancement in the breast **(A)** and ileocecal region **(B)** with enhancement **(C, D)**. Magnetic resonance imaging (MRI) reveals an iso-intensity signal compared to the muscle on T1-weighted imaging (T1WI) **(E)**, a slightly high signal on T2-weighted imaging (T2WI) **(F)** and T2-fat-suppressed (T2FS) **(G)**, and high signal on diffusion-weighted imaging (DWI) **(H)**.

**Figure 2:**
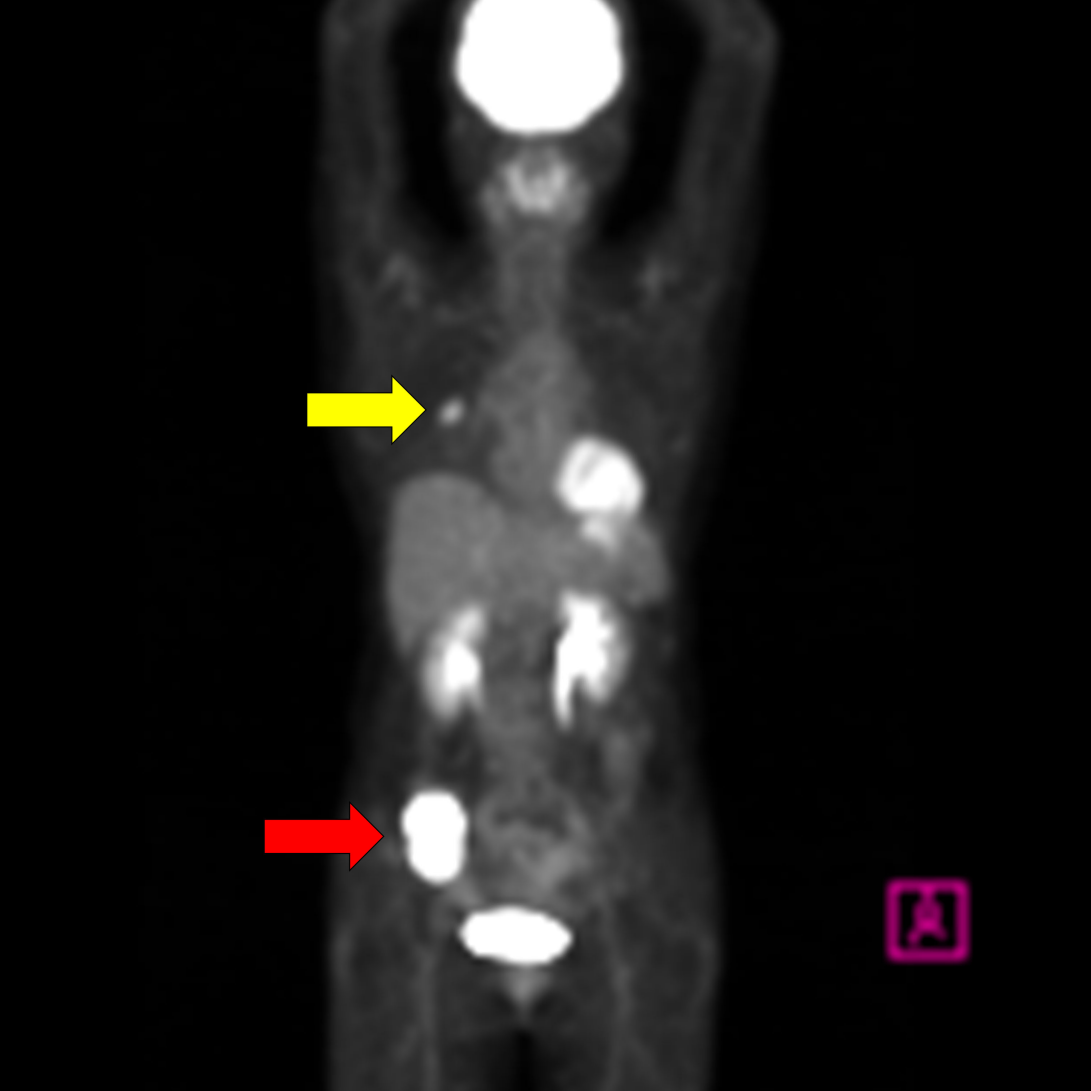
PET-CT shows a slightly high ^18^F-fluorodeoxyglucose uptake in the right breast with a maximum standardized uptake value (SUVmax) of 4.1 (yellow arrowhead) and in the ileocecal region with SUVmax of 36.9 (red arrowhead).

A 58-year-old woman noticed a lump in her right breast 5 months before the examination, and her breast mass was diagnosed as breast cancer in another hospital. Computed tomography (CT) showed an abdominal mass in the ileocecal region, and she was referred to our hospital for surgery (**Table 1**). Contrast-enhanced CT showed an enhanced mass in the right breast, diagnosed as breast cancer (**Figure 1A**). CT showed a smooth, lobulated tumor in the ileocecal region, almost iso-density to the muscle without fat or calcification (**Figure 1B**). Contrast-enhanced CT showed enhanced mass and only a small central part with contrast defect, which suggested slight degeneration (**Figures 1C, D**). The tumor was considered to be located under the mucosa (**Figures 1B–D**). Magnetic resonance imaging (MRI) showed the mass with an iso-intensity signal on T1-weighted images (WI) compared with the muscle (**Figure 1E**), slightly high signal on T2-weighted imaging (T2WI) (**Figure 1F**) and T2-weighted fat-suppressed imaging (**Figure 1G**), and high signal on diffusion-weighted imaging (**Figure 1H**). PET-CT showed slightly high ^18^F-fluorodeoxyglucose uptake in the right breast and the ileocecal region with maximum SUV (SUVmax) of 4.1 (**Figure 2**, yellow arrowhead) and 36.9 (**Figure 2**, red arrowhead), respectively. No other regions with uptake were noted. Collectively, the ileocecal tumor was suspected as GIST and lymphoma; hence, the operation was performed together for the breast and ileocecal tumors.

**Table 1:** History of present illness in the patient.

X year: Her breast mass was diagnosed as breast cancer, and CT showed the mass in both the breast and the ileocecal region
X year + 1 month: She was referred to our hospital
X year + 3 months: Operation was performed together for breast and ileocecal tumor
X year + 4 months: Hormone therapy was started postoperatively
X + 5 years + 1 month: CT showed the mass in the adrenal gland
X + 5 years + 3 months: CT-guided biopsy was performed, and the mass was diagnosed as metastasis of DDLPS
X + 5 years + 4 months: Chemotherapy was started
X + 8 years + 7 months: Liver metastasis was confirmed
X + 8 years + 11 months: Lung metastasis was confirmed
X + 9 years + 7 months: Chemotherapy was discontinued, and best supportive care was started

CT, computed tomography; DDLPS, dedifferentiated liposarcoma.

**Figure 3:**
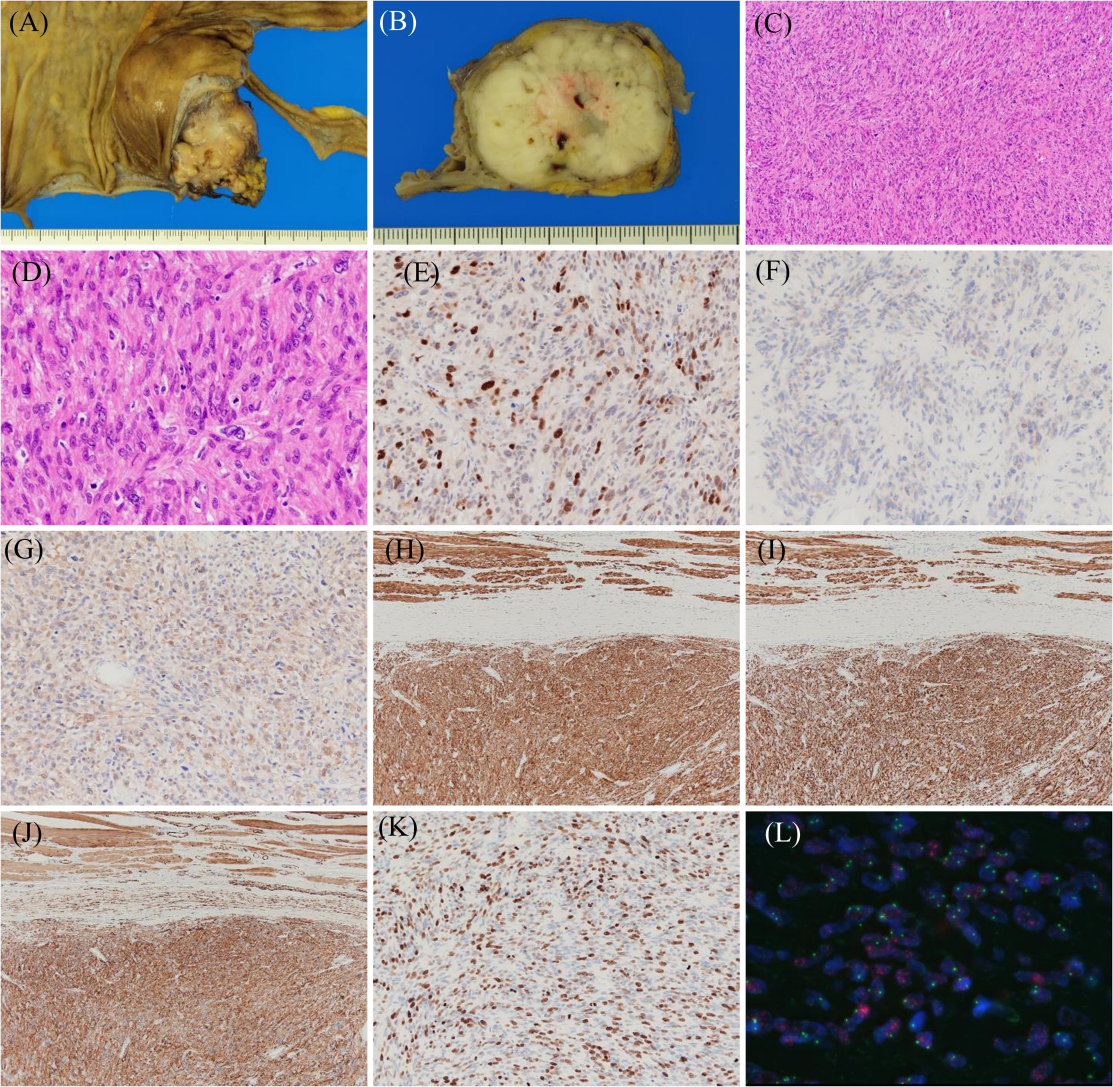
Histopathological findings of dedifferentiated liposarcoma showing a leiomyosarcoma phenotype. Resected specimens demonstrate that the tumor with lobulated and gray-white color **(A, B)** has spindle cells with eosinophilic cytoplasm and mitotic figures **(C, D)**. Immunohistochemical results for the indicated proteins are shown: **(E)** MDM2, **(F)** CDK4, **(G)** S-100, **(H)** h-caldesmon, **(I)** desmin, **(J)** α-SMA, and **(K)** Ki67. Fluorescence *in situ* hybridization shows MDM2 amplification **(L)**.

Macroscopically, a lobulated, gray-white tumor measuring 58 mm × 45 mm × 35 mm was observed in the subserosal layer of the appendix, with the main seat being the subserosal layer (**Figures 3A, B**). Histologically, spindle-shaped tumor cells with eosinophilic cytoplasm and nuclei of varying sizes and irregular shapes were observed to form bundles while wandering (**Figures 3C, D**). Mitotic figures were frequently observed [30/10 high power field (HPF)]. IHC results were as follows: MDM2 (+) (**Figure 3E**), CDK4 (+, weakly) (**Figure 3F**), S-100 (+, focally) (**Figure 3G**), h-caldesmon (+) (**Figure 3H**), desmin (+) (**Figure 3I**), α-smooth muscle actin (SMA) (+) (**Figure 3J**), HHF35 (+, focally), c-kit (−), discovered on GIST 1 (−), cluster of differentiation 34 (−), and Ki67 (MIB-1) (labeling index: 40%) (**Figure 3K**). FISH indicated MDM2 amplification (**Figure 3L**). Based on these findings, the final diagnosis of DDLPS showing leiomyosarcoma phenotype with Fédération Nationale des Centre de Lutte Contre le Cancer (FNCLCC) Grade 3 was made, and the surgical margin was negative, estimated as R0 resection. In addition, histopathological findings showed estrogen receptor (+), progesterone receptor (+), human epidermal growth factor-2 (1+), and negative margin in breast cancer. Hormone therapy was initiated post-surgery.

CT showed the mass in the adrenal gland 5 years post-surgery (**Table 1**). The CT-guided biopsy was performed to clarify which mass metastasized to the adrenal gland. The mass in the adrenal gland was diagnosed as DDLPS metastasis. Chemotherapy was administered for 3 years, and unfortunately, the metastasis of the iliopsoas muscle, liver, and lung had developed. She decided to discontinue the chemotherapy and was treated with the best supportive care.

## 3 Discussion

DDLPS is a subtype of liposarcoma; it frequently occurs in the retroperitoneum (1). Retroperitoneal dedifferentiation from ALT/WDLPS sometimes occurs in approximately 10% of cases, and the fat component is enough to generate suspicious findings for DDLPS and ALT/WDLPS (3, 4). ALT/WDLPS and DDLPS have the capability for recurrence, but only DDLPS can metastasize (3). Liposarcoma rarely occurs in the gastrointestinal tract, but among the cases that occur in the gastrointestinal tract, ALT/WDLPS and DDLPS are relatively common. However, the nature of DDLPS, including recurrence and metastasis, remains unclear because the follow-up period was short (2). GIST and lymphoma are the most common tumors in the ileocecal region, and the differential diagnosis of GIST and smooth muscle tumors, including leiomyosarcoma, nerve sheath, and fibroblastic tumors, is important (6). In our case, the occurrence of DDLPS in an extremely rare location, coupled with two factors (imaging and pathological findings), made the diagnosis more difficult. In the next section, we highlight how the two factors were complex.

First, MRI and PET findings were tricky. DDLPS is easily suspected in a case involving the retroperitoneum (typical location) and the presence of a fat component. Based on the proportion of fat components, Yun et al. classified DDLPS MRI findings into four types (4). Category 3, having low-fat content, accounts for 24%, and Category 4, which has almost no fat content, accounts for 44% (7). In Category 3, preoperative diagnosis by MRI mainly indicates soft tissue sarcoma, and another differential diagnosis is DDLPS. Unfortunately, in Category 4, preoperative diagnosis is “non-specific”, and a further differential diagnosis cannot be made (4). In addition, spindle cell lipoma and atypical spindle cell/pleomorphic lipomatous tumors, which are benign, showed various levels of fat component, suggesting that MRI findings in these benign tumors are similar to those in DDLPS (8). Next, regarding PET, SUVmax in DDLPS significantly increased compared to that in ALT/WDL (average of 6.2 vs. 2.8), which suggested that SUVmax can differentiate between ALT/WDLPS and DDLPS. Furthermore, SUVmax correlated with FNCLCC Grade (5). In this case, the SUVmax on PET was extremely high (36.9). The tumor originated from a mesenteric or submucosal lesion in CT. Based on these findings, GIST and lymphoma were most likely diagnosed preoperatively.

In our case, imaging and histopathological findings were tricky. The differential diagnosis, even for the usual DDLPS, is wide-ranging, reflecting the direction and degree of differentiation (3). Generally, image-guided biopsy is recommended for diagnosing retroperitoneal sarcoma (9). In our case, the mass in the ileocecal region was suspected to be malignant because of a high SUV max on PET. However, we considered the following factors: i) the feasibility of R0 surgery, ii) the patient’s preference for a one-stage resection, and iii) the risk of tumor dissemination from biopsy. As a result, a biopsy was not performed. Careful diagnosis should be made in differentiating liposarcoma, including pleomorphic and myxoid liposarcoma, and other tumors, including solitary fibrous tumors, GIST, synovial sarcoma, and malignant peripheral nerve sheath tumors, even if IHC and FISH were performed (3). In addition, 5%–10% of DDLPS occurred in the heterogeneous differentiation with osteoid, chondroid, or myoid (1). We suspected leiomyosarcoma in this case because of positive SMA and h-caldesmon. IHC for MDM2 and CDK4 and FISH for MDM2 are powerful tools for correct diagnosis. The IHC results for MDM2 and CDK4 were negative in leiomyosarcoma and positive in DDLPS in over 90% of the cases (3, 10). Recently, the roles of CDK4 have been reported as follows: i) high CDK4 alterations occur in DDLPS among soft tissue sarcomas, ii) CDK4 alteration correlates with worse overall survival, and iii) the levels of CDK4 and MDM2 are not always consistent (11). SMA and h-caldesmon, muscle markers, were positive for leiomyosarcoma and DDLPS with leiomyosarcoma phenotype (10). Furthermore, whether myogenic differentiation in DDLPS affects recurrence and prognosis is controversial, and further analysis is needed (10, 12). Whole DDLPS and FNCLCC Grade correlated with the overall survival (OS) and distant metastasis in DDLPS with myogenic differentiation (12).

The primary strategy for treatment is surgery. R0 and R1 resections were correlated with a lower recurrence rate and longer prognosis compared to R2 resection (13). However, organs close to the tumor, such as the kidney, large intestine, and ureter, may sometimes be resected, leading to deterioration of the patient’s condition (13). The combination of radiotherapy and chemotherapy with surgery has been expected to avoid this situation. Preoperative radiotherapy contributed to local control in Grade 1 and 2 DDLPS. However, it did not affect OS. Conversely, neither local control nor OS in the Grade 3 DDLPS had any impact, suggesting that the efficacy of radiation for DDLPS was limited (14). In addition, radiotherapy was not performed in our case because the surgical margin was R0, and the DDLPS was evaluated as Grade 3. In recent years, clinical trials of inhibitors targeting MDM2-P53 and CDK4/6 have been conducted (15). There have been several recent reports on the CDK4/6 inhibitor palbociclib, although well tolerated, showing limited efficacy as monotherapy. This suggests that combination therapy with other agents and further exploration of potential biomarkers for CDK4 inhibitors are needed (16–18). If better efficacy is obtained in the clinical trials, various combination therapies, for example, reduction surgery with chemotherapy and radiation, and chemotherapy and radiation for inoperable cases, may be developed. The target points of these inhibitors are MDM2 and CDK4, and the role of correct diagnosis of DDLPS by histopathological findings will become much larger.

## 4 Conclusion

In conclusion, we report an extremely rare case of DDLPS in the ileocecal region with leiomyosarcoma phenotype. Although our case is atypical because of the presence of double cancer, we adhered to the general treatment process for carcinoma and sarcoma, which was carried out after pathological examination through biopsy and imaging findings. In addition, DDLPS occasionally shows heterogeneous differentiation, highlighting the need for extra caution because of the possibility of misdiagnosis. Taken together, accurate diagnosis through IHC and FISH for MDM2 and CDK4 may play crucial roles not only in differential diagnosis but also in treatment decision-making.

## Data Availability

The original contributions presented in the study are included in the article/supplementary material. Further inquiries can be directed to the corresponding author.
